# Reply to: No new evidence for an Atlantic eels spawning area outside the Sargasso Sea

**DOI:** 10.1038/s41598-022-14885-5

**Published:** 2022-07-11

**Authors:** Yu-Lin K. Chang, Eric Feunteun, Yasumasa Miyazawa

**Affiliations:** 1 Application Laboratory, Japan Agency for Marine-Earth Science and Technology, Yokohama, 236-0001 Japan; 2 Museum National d’Histoire Naturelle, BOREA (MNHN, CNRS, SU, IRD, UCN, UA), Station Marine de Dinard, 35800 Dinard, France

**Keywords:** Marine biology, Animal migration

**replying to**: H. Reinhold et al.; *Scientific Reports* 10.1038/s41598-022-14882-8 (2022).

## Introduction

The Sargasso Sea has long been considered as the spawning area for Atlantic eels, despite the absence of direct observations after more than a hundred years of the survey. We proposed a new insight on the location of Atlantic eels spawning areas eastward of the Sargasso Sea at the intersection between the Mid-Atlantic Ridge and the oceanic fronts^1^. Our hypothesis is based on a body of corroborating cues from literature. We suggested that European silver eels converge towards the Azores whatever their departure point from Europe and Northern Africa, then they follow the Mid-Atlantic Ridge south westerly until they reach oceanographic fronts where temperature and depths are favourable for reproduction. These orientation behaviours are potentially based on magnetic fields and odours that might be generated by the Mid-Atlantic Ridge volcanic activity and detected by eels during their diel vertical movements. The first favourable meeting point is then located at the crossing between the Mid-Atlantic Ridge and the oceanic thermic isotherms located around 45° W and 26° N. Our hypothesis is supported by (i) microchemical differences between the core of otoliths extracted from leptocephali collected in the Sargasso Sea and from glass eels collected across Europe suggesting that glass eels hatch in different chemical environments than leptocephali (ii) an asymmetric genetic introgression between American and European eels^2^ suggesting that the overlapping spawning areas favour transport of hybrids towards northern Europe rather than to America and to southern Europe. This supports the possible existence of several distinct spawning areas, where currents favour transport either westward (American eel), north eastward (hybrids and European eels) or eastward (European eels). To test this hypothesis, we developed a transport model and compared the dispersion dynamics of virtual leptocephali released from the Sargasso Sea and from above the Mid-Atlantic Ridge. The transport models showed that virtual eels released from the Mid-Atlantic Ridge reached Europe and America following similar patterns than those released from the Sargasso Sea thus supporting the Mid-Atlantic Ridge spawning hypothesis.

**Figure 1:**
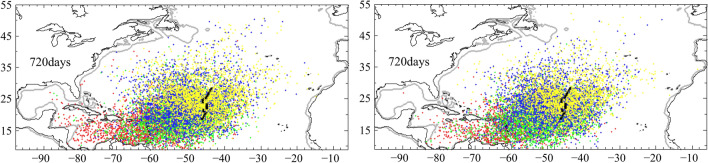
Distribution of v-larvae released departure at the west (left) and east (right) of Mid-Atlantic Ridge. The tracking method is the same as described in the paper, v-larvae were release within 100 km west and east of the ridge.

Hanel et al.^3^ have raised several concerns, one of which being that “microchemical evidence was the only was the major argument supporting the Mid-Atlantic Ridge hypothesis”. This was their start point of a critical rebuttal of our findings to question our hypothesis. Instead, we consider that our regrettable error does not fundamentally contradict the possibility that eels do indeed successfully spawn outside the so-called Sargasso Sea.

(Comment 1) The importance of seamounts as orientation and navigation cues towards a spawning area was hypothesized, no clear mechanism is proposed for how the migrating eels can detect the ridge.

(Response 1) Our Hypothesis does not state that eels find a kind of shallow seamount where they spawn. Instead, we propose that orientation of silver eels during their spawning migration could be based on a combination of behavioural mechanisms including geomagnetism, odours, temperature and salinity gradients^4–8^. These environmental cues and related gradients are strongly controlled or influenced by the topography of the oceanic floor. The Mid-Atlantic Ridge and the Mariana areas have similarities with ridges and seamount chains oriented perpendicularly to temperature and salinity fronts surrounded by deep abyssal plains. Our Mid-Atlantic Ridge hypothesis proposed that Atlantic eels could use similar signposts as Japanese eel, which hatch near the Mariana Ridge^9^. Indeed, as for the Japanese eels, the orientation mechanism that lead Atlantic eels from the growth areas to the ridge are not understood, but the empirical observations from Righton et al.^10^ suggest that eels converge towards the Azores whatever their release point across Europe and that their diel vertical migration takes them down to 500–1000 m every day. The reasons for this behaviour are not elucidated, but since they cost energy, they are likely compensated by advantages such as orientation together with predator avoidance and sexual maturation^11–13^. Following our hypothesis, eels search for orientation cues during DVM. The geomagnetic fields are suggested to provide detectable information for silver eels on their oceanic spawning migration^14^. However, whether magnetic characteristics of the Mid-Atlantic ridge may provide detectable orientation cues still needs to be documented. Similarly, the existence of detectable odours that might be generated by the tectonic activity and hydrodynamics of the Mid-Atlantic ridge and serve as orientation cues for eels is still unknown. Hydrodynamic mesoscale turbulence and vertical flows have been shown to be generated along the Mid-Atlantic Ridge^15^, which we propose eels might be able to detect. There are no well supported spawning areas of freshwater eels other than *A. japonica* and one north Pacific population of *A. marmorata*. The spawning areas of the other species remain unknown. In the south west Indian Ocean, spawning areas of 3 species (*A. mossambica*, *A. marmorata* and *A. bicolor*) were proposed on the east of the Mascarene Ridge with a similar topography (although shallower) than along the Mid-Atlantic Ridge and the Mid-Pacific ridge and seamounts^16,17^. Inaccurate spawning areas were also proposed for the South Pacific *A. diffenbachii* between Fiji, New Caledonia and New Zealand; in the vicinity of a number of oceanic ridges and trenches^18^ that may also serve as landmarks. Because all eel species studied on their spawning migration show similar diel vertical migration behaviours, it is likely that common orientation mechanisms could lead to detection of oceanographic variability related to the topography of the sea floor and related geomagnetism, local hydrodynamic turbulence and odour caused by vertical currents. This kind of oceanic landscape (chains of seamounts) occurs on narrow areas which strongly increase the meeting probability of spawners searching for partners and favourable spawning places.

(Comment 2) Drift simulation with departures from the Mid-Atlantic Ridge and from the Sargasso Sea showed similar results. This is not surprising since the modelling of larval drift seems essentially just to reflect the slow westward drift prevailing both in the Sargasso Sea and Mid-Atlantic Ridge areas. The assumption of using the intersection of the Mid-Atlantic Ridge by the two thermal fronts as presumed spawning places seems to have little basis. There is no indication neither of one nor two temperature fronts at depths where leptocephali are found along a 45  W latitudinal section in the middle of the Mid-Atlantic Ridge area.

(Response 2) We agree with the comments that the similar distributions between the departure from the Sargasso Sea and the Mid-Atlantic Ridge are expected, as they mainly reflect the ocean circulation. This is also what we wanted to address, if different departures could lead to similar distributions, either Sargasso Sea or Mid-Atlantic Ridge could be candidates for the spawning area. We also agree that many eel larvae were collected at the two fronts in the Sargasso Sea, but not near the Mid-Atlantic Ridge. However, if the departure from the Sargasso Sea and the Mid-Atlantic Ridge led to similar distributions after 720 days, they were not the result of westward current, but the cause of a relatively quiet ocean in the Sargasso Sea and its surrounding area (i.e. Fig. 1). Without prevailing current, small larvae were mainly transported by ocean dispersion, and would later be transported by the major currents that lie in the north (Azores Current), south (North Equatorial Current), and west (Gulf Stream) of the Sargasso Sea. So, we compared departures at 100 km from west and east of the Mid-Atlantic Ridge. Subtle differences occurred (figure below). V-larvae departing from the east of the ridge dispersed relatively less northward compared to larvae released 100 km at the west of the ridge (this figure and original paper). Secondly v-larvae released at the south east of the study area (red dots on the figure, right panel) disperse relatively less towards the Caribbean Sea than when released at the west (red dots of the figure, left panel). This suggests that the dispersion of European eel larvae is optimum in an area comprised between the Mid-Atlantic Ridge and the Sargasso Sea (our previous simulation in the original paper), and declines eastward of the Mid-Atlantic Ridge (present simulation below).

Hanel et al. also indicate that the convergence front weakens from West, in the Sargasso Sea, to East above the ridge. We consider that this constitutes an additional argument that the Mid-Atlantic Ridge is indeed at the edge of the convergence zone at the first area of the Atlantic Ocean where currents and temperatures are favourable for reproduction of eels.

(Comment 3) Elevated manganese (Mn) concentrations in the otolith cores of glass eels as a hint for successful spawning only in areas with volcanic activity based on observations of Martin et al.^18^. However, the results from Martin et al.^19^ were entirely misread, resulting in a mis-interpretation of the data.

(Response 3) Based on Martin et al.^19^, we stated that higher concentrations of Mn were found in glass eels’ otoliths collected across European estuaries than in otoliths of leptocephali larvae sampled in the Sargasso Sea. We suggested that this was the indication that glass eels were born in areas where volcanic activity produces high loads of Mn and other metals. This formed one of the arguments supporting our hypothesis that Atlantic eels could spawn in the proximity to the Mid-Atlantic Ridge. Thanks to Reinhold Hanel and colleagues, we realized that Martin et al.^19^ in fact showed that concentrations of Mn were higher in the center of otoliths of leptocephali larvae than in those of glass eels collected along the European coasts. Consequently, this argument is no longer valid. Nonetheless, otolith microchemical fingerprints significantly differ between young leptocephali sampled in the Sargasso Sea in 2008 and glass eels collected in Europe, hence suggesting that they have distinct spawning areas^19^. These authors indicated that the incorporation of elements from the environment to the otoliths needed to be better understood, namely as stated by Hanel et al., because of physiological and environmental control such as temperature and salinity. In addition, they outline that the dynamics of elements from the sea floor to the subsurface is not well understood and could be slow. We totally share these conclusions that are well known facts, and that simply confirm that environmental characteristics (trace element concentrations, salinity and temperature) are responsible for the elemental signature of the central part of otoliths. Hanel et al. also state that the composition of otoliths are also controlled by elemental maternal transfer from the egg to the otoliths. We are aware of this fact that has been shown is other fish species. However, the laser ablations were performed after the first feed check where maternal influence is reduced and is overruled by environment^18^. This supports the idea that glass eels collected in Europe do not originate from the same environments as leptocephali captured in the Sargasso Sea.

(Comment 4) Insufficient sampling efforts and a limited area coverage of recent surveys as a possible reason for “false negative” observations along the Mid-Atlantic Ridge. This statement does not recognize the investigations by Johannes Schmidt as well as earlier and later surveys in the Mid-Atlantic Ridge area. The ICES “Eggs and Larvae database” records a total of 48 *A anguilla* leptocephali caught within the area 15–29 N and 43–48 W, at 10 stations between 1913 and 1970.

Thanks for pointing out that larvae have been caught near the Mid-Atlantic Ridge, in which larvae were not newly hatched because of their relatively large size (23–45 mm). Ocean currents were weak and could flow either eastward or westward in this region, indicating that the spawning could occur from west to east of the ridge, without considering swimming. Note that ocean currents could change directions, so that it was also possible to spawn near the ridge after been transported eastward and westward.

The observed distribution of small larvae < 10 mm extends over a wide area (1500 ~ 2000 km)^20^ confirms that Atlantic eels do spawn in the so called Sargasso sea. We argue that the lack of small larvae along the Mid-Atlantic Ridge could be a result of a much lower sampling effort and a different timing than in the Sargasso Sea, while the absence of occurrence does not form a proof that eels do not spawn over the ridge. Again, we propose that the spawning area could extend eastward, and we propose to extend surveys to the east over the Mid-Atlantic Ridge to test our hypothesis. It will be interesting to give the opportunity (again) to explore the Mid-Atlantic Ridge region. Modern technology that has recently progressed may enable new findings.

## Conclusion

Our initial article^1^ has initiated an innovative reflection to narrow the area investigated to find eels’ spawning areas. To this end, we have proposed a theoretical framework to explore likely orientation and meeting mechanisms of eels on their spawning migration to the spawning areas. None of the arguments and models we propose thoroughly prove that spawning exists outside the huge Sargasso Sea, but they all converge (including genetic studies shortly discussed in the introduction) to suggest that spawning occurs in multiple places in the Sargasso Sea and that spawning over the Mid-Atlantic Ridge is possible, if not likely. We hope that this new hypothesis contributes to stimulate research on the spawning migration and areas of the European eels. Moreover, sampling eel larvae and planktonic food-web over the Mid-Atlantic ridge would provide new insights on the global functioning of the North Atlantic Ocean, thus mutualising the costs of research cruises for multidisciplinary approaches.

## References

[1] Chang YK (2020). New clues on the Atlantic eels spawning behavior and area: The Mid-Atlantic Ridge hypothesis. Sci. Rep..

[2] Wielgoss S (2014). Introgressive hybridization and latitudinal admixture clines in North Atlantic eels. BMC Evol. Biol..

[3] Hanel R, Marohn L, Westerberg H (2022). No new evidence for an Atlantic eels spawning area outside the Sargasso Sea. Sci. Rep..

[4] Naisbett-Jones LC (2017). Magnetic map leads Juvenile European Eels to the Gulf Stream. Curr. Biol..

[5] Durif CMF (2013). Magnetic compass orientation in the European Eel. PLoS ONE.

[6] Pankhurst NW, Lythgoe JN (1983). Changes in vision and olfaction during sexual maturation in the European eel *Anguilla anguilla* (L.). J. Fish Biol..

[7] Barbin GP, Parker SJ, McCleave JD (1998). Olfactory clues play a critical role in the estuarine migration of silver-phase American eels. Environ. Biol. Fishes.

[8] Churcher AM (2015). Deep sequencing of the olfactory epithelium reveals specific chemosensory receptors are expressed at sexual maturity in the European eel *Anguilla anguilla*. Mol. Ecol..

[9] Tsukamoto K (1992). Discovery of the spawning area for Japanese eel. Nature.

[10] Righton D (2016). Empirical observations of the spawning migration of European eels: The long and dangerous road to the Sargasso Sea. Sci. Adv..

[11] Wahlberg M (2014). Evidence of marine mammal predation of the European eel (*Anguilla anguilla* L.) on its marine migration. Deep Sea Res. Part..

[12] Trancart T (2014). The effect of thermal shock during diel vertical migration on the energy required for oceanic migration of the European silver eel. J. Exp. Mar. Biol. Ecol..

[13] Westerberg H (2021). Predation on migrating eels (*Anguilla anguilla* L.) from the Western Mediterranean. J. Exp. Marine Biol. Ecol..

[14] Durif CM (2021). A unifying hypothesis for the spawning migrations of temperate anguillid eels. Fish Fish..

[15] Lahaye N (2019). Deep currents in the rift valley of the north mid-atlantic ridge. Front. Mar. Sci..

[16] Pous S, Feunteun E, Ellien C (2010). Investigation of tropical eel spawning area in the South-Western Indian Ocean: Influence of the oceanic circulation. Prog. Oceanogr..

[17] Robinet T, Réveillac E, Kuroki M, Rabenevanana MW, Valade P, Gagnaire PA, Aoyama J, Berrebi P, Tsukamoto K, Feunteun E (2008). New clues for freshwater eels (*Anguilla* spp.) migration routes to eastern Madagascar and surrounding islands. Marine Biol..

[18] Watanabe S, Aoyama J, Tsukamoto K (2004). Reexamination of Ege’s (1939) use of taxonomic characters of the genus anguilla. Bull. Mar. Sci..

[19] Martin J (2010). An otolith microchemistry study of possible relationships between the origins of leptocephali of European eels in the Sargasso Sea and the continental destinations and relative migration success of glass eels. Ecol. Freshw. Fish.

[20] Miller MJ (2015). A century of research on the larval distributions of the Atlantic eels: A re-examination of the data. Biol. Rev..

